# Apalutamide versus bicalutamide in combination with androgen deprivation therapy for metastatic hormone sensitive prostate cancer

**DOI:** 10.1038/s41598-024-51389-w

**Published:** 2024-01-06

**Authors:** Takashi Ueda, Takumi Shiraishi, Masatsugu Miyashita, Naruhiro Kayukawa, Yusuke Gabata, Satoshi Sako, Ryota Ogura, Atsuko Fujihara, Koji Okihara, Osamu Ukimura

**Affiliations:** 1 Department of Urology, Graduate School of Medical Science, Kyoto Prefectural University of Medicine, Kyoto, Kyoto 602-8566 Japan; 2 Department of Urology, Maizuru Kyosai Hospital, Maizuru, Kyoto 625-8585 Japan

**Keywords:** Prostate, Cancer therapy

## Abstract

The objective of this study is to compare the efficacy of apalutamide and bicalutamide in combination with androgen deprivation therapy in patients with metastatic hormone-sensitive prostate cancer (mHSPC). We retrospectively collected the data of about 330 patients with metastatic hormone-sensitive prostate cancer at our hospital and affiliated hospitals between December 2013 and August 2023. Sixty-one patients were administered apalutamide (240 mg/day) with androgen deprivation therapy (group A), and 269 patients were administered bicalutamide (80 mg/day) with androgen deprivation therapy (group B). Propensity score matching was used to adjust for clinical background factors between the two groups. PSA progression-free survival and overall survival were significantly longer in group A than in group B among the matched patients. Apalutamide therapy was a significant independent factor for OS in matched patients. The second progression-free survival of group A was significantly longer than that of group B in matched patients. Patients treated with apalutamide achieved ≥ 90% PSA decline from baseline faster and in larger numbers than those with bicalutamide. Apalutamide combined with ADT may be superior to bicalutamide alone in terms of OS and PSA-PFS in patients with mHSPC.

## Introduction

Apalõutamide, a second-generation androgen receptor axis-targeted agent (ARAT), has been accepted as a standard first-line drug for patients with metastatic hormone-sensitive prostate cancer (mHSPC), since the TITAN trial demonstrated that apalutamide plus androgen deprivation therapy (ADT) significantly improved the overall survival (OS) in patients with mHSPC compared to that by placebo plus ADT^1^. Although combined androgen blockade (CAB) therapy using first-generation antiandrogens, such as bicalutamide, is not recommended as a first-line drug for patients with mHSPC in the National Comprehensive Cancer Network and European Association of Urology guidelines, CAB therapy is recommended as one of the standard first-line therapies in the Japanese clinical practice guidelines for prostate cancer^2^. According to the study using Japanese prostate cancer database, CAB therapy resulted in significantly better overall survival and cancer specific survival compared to non-CAB therapy such as ADT monotherapy in patients with high Gleason score and prostate-specific antigen level and advanced clinical stage^3^. Therefore, information comparing the effectiveness of apalutamide plus ADT and CAB therapy could be useful in Japanese clinical practice for patients with mHSPC. Though several previous reports compared the efficacy of ARATs including apalutamide with that of bicalutamide^4,5^, they were not direct comparison of the efficacy between apalutamide and bicalutamide.

Several prognostic indicators of long-term outcomes, such as OS, have been reported^6,7^. Second progression-free survival (PFS2), the time duration from the date of first-line therapy to the progression of second-line therapy, has been suggested as a prognostic indicator of patients with mHSPC^8^. In the TITAN trial, PSF2 was assessed as a clinically relevant endpoint, and PFS2 was longer in patients with mHSPC treated with apalutamide than in those treated with placebo^1^. Prostate-specific antigen (PSA) kinetics, such as the percentage of PSA decline from baseline, have been reported as prognostic indicators of OS in patients with mHSPC^6,7,9,10^. The OS of patients with mHSPC who achieved a deep PSA decline (≥ 90% PSA decline) was longer than that of patients with mHSPC who did not.

The purpose of this study was to compare the efficacy of apalutamide with that of bicalutamide in combination with ADT for mHSPC, and to observe the correlation of prognostic indicators with OS in patients with mHSPC treated with apalutamide.

## Methods

### Patients and treatments

We retrospectively collected data about 330 patients with mHSPC treated at our hospital and affiliated hospitals between December 2013 and August 2023. Sixty-one patients were administered apalutamide (240 mg/day) with androgen deprivation therapy (group A), and 269 patients were administered bicalutamide (80 mg/day) with androgen deprivation therapy (group B).

Bone and visceral metastases were assessed using bone scintigraphy and computed tomography (CT). Common Terminology Criteria for Adverse Events (CTCAE) were used to assess the severity of adverse events. Drug dose reduction in case of adverse events was performed according to the instructions provided by pharmaceutical companies.

Two consecutive increases in PSA of 50% compared with nadir and ≥ 2 ng/ml on two consecutive measurement at least 1 week apart were defined as PSA recurrence.

This study was approved by the Institutional Review Board of Kyoto Prefectural University of Medicine (ERB-C-1071-2) and each affiliated hospital, and was conducted in compliance with the Declaration of Helsinki. The institutional review board waived the requirement for individual written informed consent owing to the retrospective nature of this study. Opt-out information was provided to patients on the website.

### Statistical analysis

We used the chi-square and Wilcoxon rank-sum tests to compare the two groups, as appropriate. We used Kaplan–Meier analysis to estimate the differences in time events between the two groups using the log-rank test. Cox proportional hazard models were used to investigate factors associated with PFS. Propensity score matching was used to adjust for the clinical background between the two groups. Statistical analyses were performed using SAS JMP, Version 14, and P < 0.05 was taken to indicate statistical significance.

## Results

### Adjustment of clinical background of the patients by propensity score matching

The clinical background of the cohort is presented in Supplementary Table 1. Sixty-one patients were treated with apalutamide or ADT (group A). A total of 269 patients were treated with bicalutamide and ADT (Group B). Information regarding the adverse events is shown in Supplementary Table 2. Twelve (20%) grade 3 skin disorders were diagnosed based on the Common Terminology Criteria for Adverse Events version 5.0 Eight (13%) patients in Group A and 156 (58%) patients in Group B were treated with sequential drugs after disease progression, as shown in Supplementary Table 3. There were no significant differences in age, Performance status (PS), pretreatment PSA, Gleason score (GS), and presence of bone and visceral metastases between group A and group B (p = 0.8375, 0.4231, 0.1591, 0.3639, 0.6520, and 0.1230, respectively). Pretreatment alkaline phosphatase (ALP) levels and the presence of lymph node metastasis in group B was significantly higher than in group A (p = 0.0032 and p = 0.00901, respectively). The observation period in group A was significantly shorter than that in group B (< 0.0001). To adjust for clinical background factors between Groups A and B, we used propensity score matching technique. The clinical backgrounds of the 104 matched patients are shown in Table 1.

**Table 1:** Patient characteristics of matched patients.

Hormone therapy		Apalutamid + ADT (group A)	Bicalutamide + ADT (group B)	A vs B
		n = 52	n = 52	p-value
Median age at diagnosis years (range)		77.5 (61–87)	80 (64–94)	0.2115
Performance status (ECOG)	0	37	31	0.4448
	1	10	15	
	2	5	6	
Median pretreatment PSA level ng/mL		104.5(1.26–7756)	143(9.8–8700)	0.8704
Median pretreatment ALP		230 (56–1682)	259 (59–1072)	0.9898
Pathological diagnosis	Gleason score 6	1	0	0.2706
	Gleason score 7	5	4	
	Gleason score 8	17	15	
	Gleason score 9	26	28	
	Gleason score 10	3	5	
Presence of bone metastasis	Yes	44	47	0.3737
	No	8	5	
	Not applicable	2	0	
Presence of visceral metastasis	Yes	16	12	0.3765
	No	36	40	
Presence of lymph node metastasis	Yes	30	27	0.5545
	No	22	25	
Median observation period month (range)		16 (3–35)	14.5 (3–35)	0.5464

ECOG, Eastern Cooperative Oncology Group; PSA, prostate-specific antigen; ALP, alkaline phosphatase; ADT, androgen deprivation therapy.

### PSA progression-free survival and overall survival observation in matched patients

**Figure 1:**
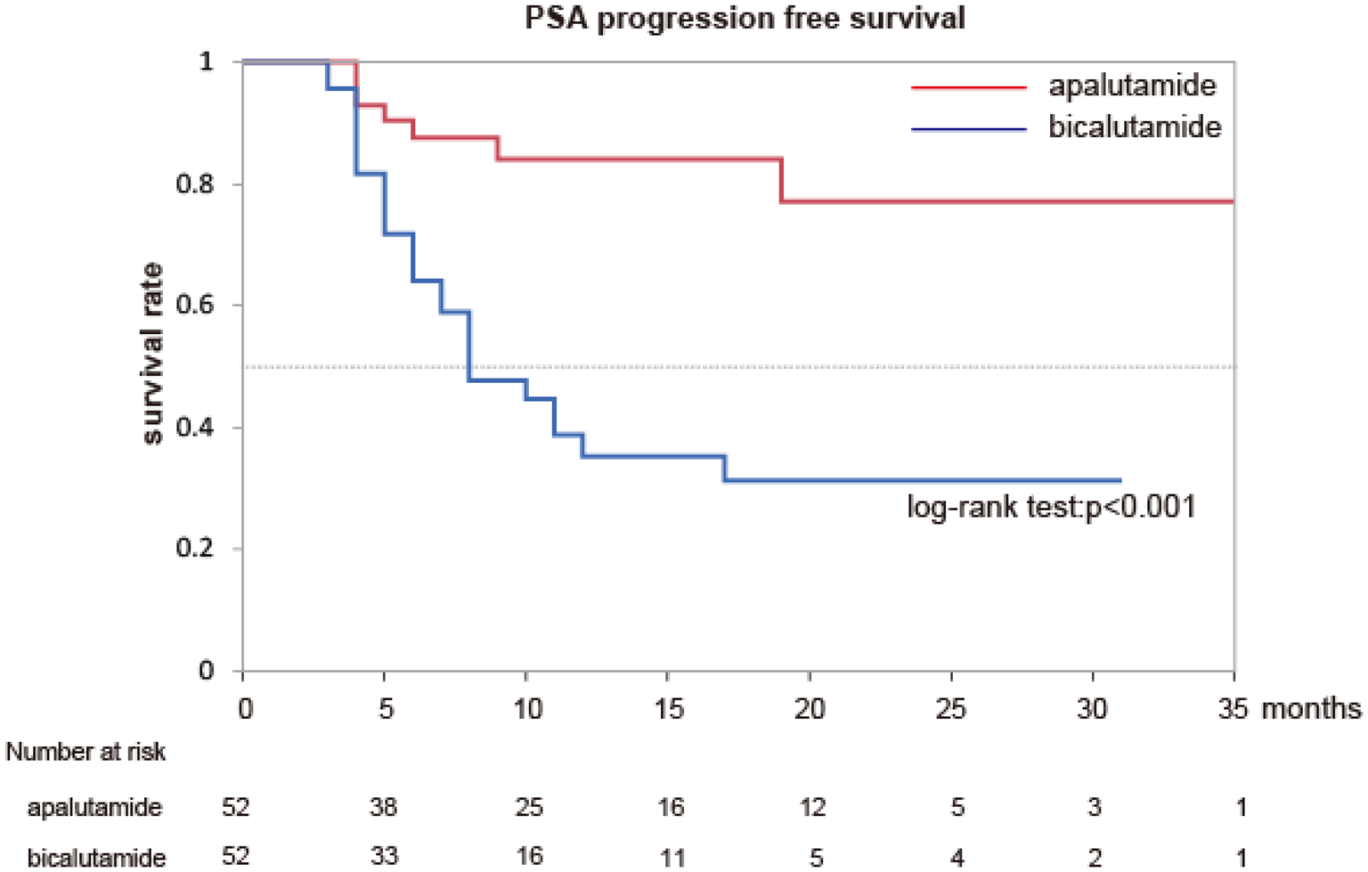
Kaplan–Meier estimates of PSA progression free survival in matched patients.

**Figure 2:**
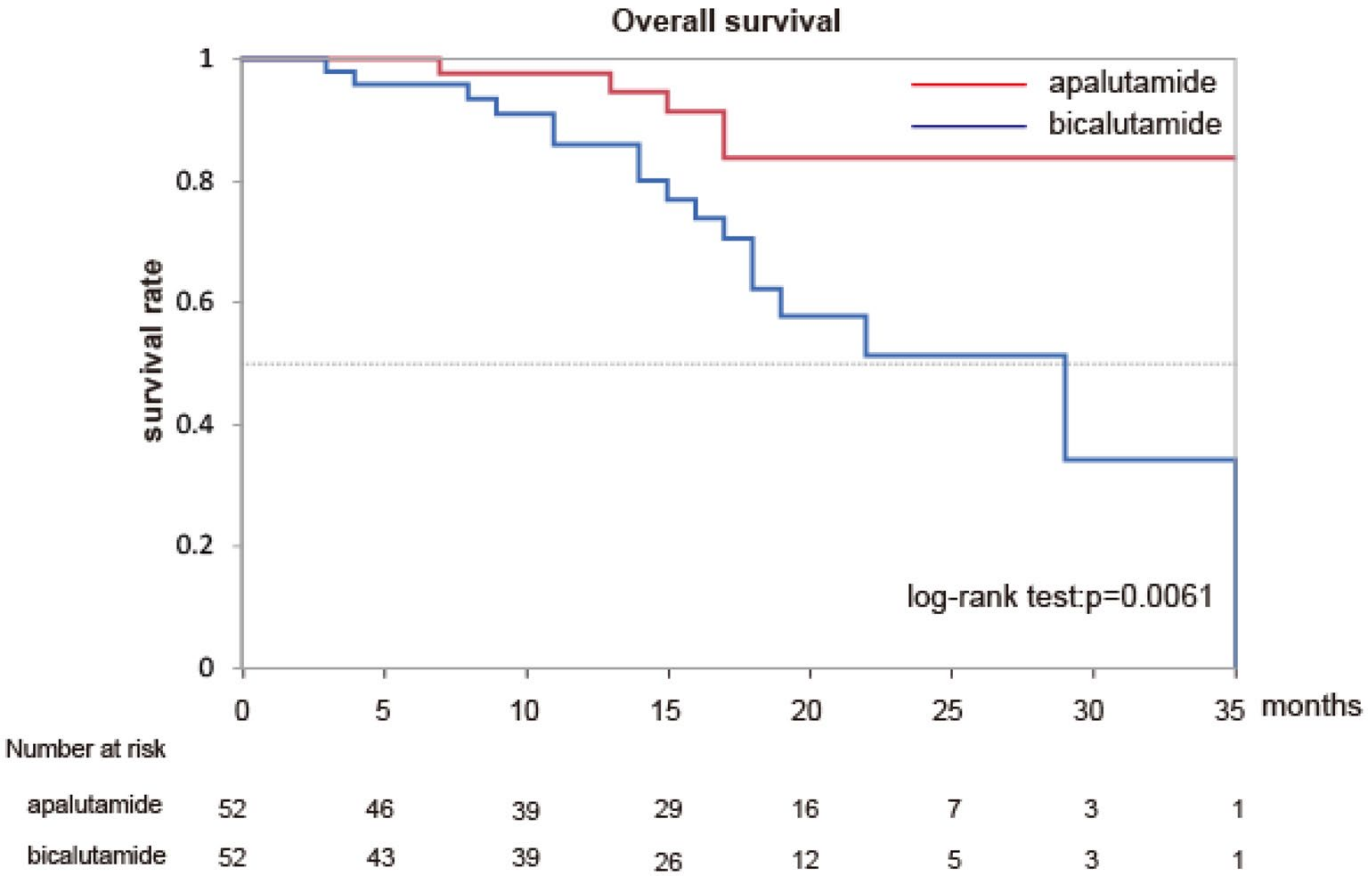
Kaplan–Meier estimates of overall survival in matched patients.

To investigate the difference in efficacy between apalutamide and bicalutamide, we observed the PSA-PFS and OS in matched patients. The PSA-PFS and OS of group A were significantly longer than those of group B (Fig. 1 (p < 0.001) and Fig. 2 (p = 0.0061)). Pre-treatment PSA and ALP levels, Gleason score, and the presence of visceral metastasis are prognostic factors for OS in mHSPC treatment^11,12^. We performed multivariate and univariate analyses using Cox Logistic regression to investigate the factors associated with OS in patients with mHSPC. We included variables for these factors and antiandrogen use (apalutamide or bicalutamide). CAB therapy was shown to be a significant independent factor for OS in high-risk patients with mHSPC in both univariate and multivariate analyses (Table. 2). These results suggest that apalutamide may be superior to bicalutamide in terms of PSA-PFS and OS for the treatment of patients with mHSPC.

**Table 2:** Univariate and multivariate analyses for the overall survival in matched patients.

	Univariate analysis			Multivariable analysis		
	HR	95% CI	p-value	HR	95% CI	p-value
CAB therapy	4.296	1.323–13.93	0.0069	3.66	1.35–9.95	0.0109
Pretreatment PSA level	29.88	2.432–310.80	0.0108	1.00	0.999–1.000	0.2319
Gleason score	4.851	3.057–25.05	0.2256	1.254	0.722–2.219	0.4271
pretreatment ALP level	9.289	0.281–118.2	0.1798	13.42	0.851–85.35	0.0616
Presence of visceral metastasis	2.982	0.686–12.96	0.1450	2.225	0.654–7.570	0.2004

HR, hazard ratio; CI, confidence interval; CAB, combined androgen blockade; PSA, prostate-specific antigen; ALP, alkaline phosphatase; LN, lymph node.

### Prognostic indicators of OS in treatment for mHSPC patients

**Figure 3:**
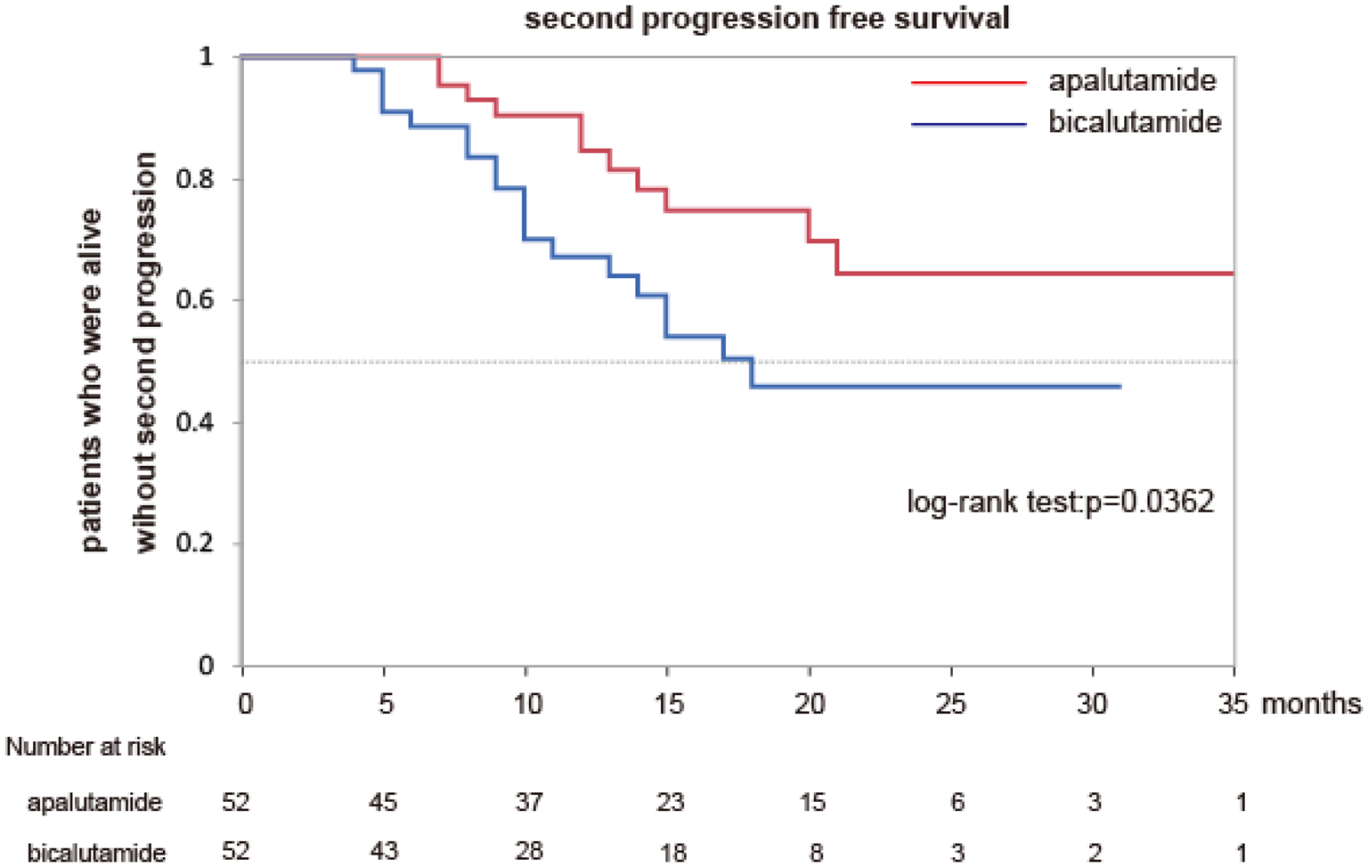
Kaplan–Meier estimates of second progression free survival in matched patients.

**Figure 4:**
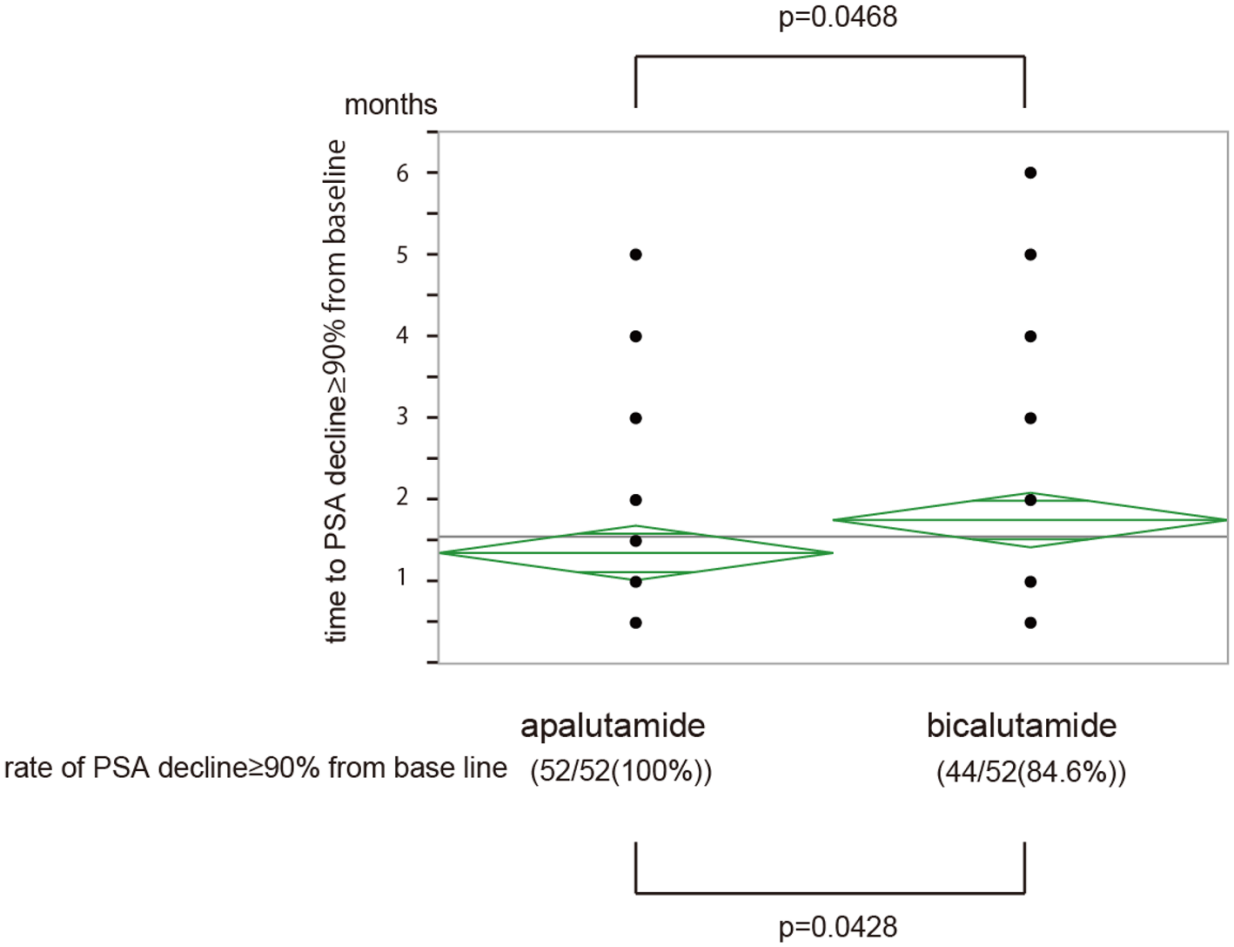
PSA kinetics in matched patients.

To determine how apalutamide prolongs the OS of patients with mHSPC compared to that by bicalutamide, we explored the prognostic indicators of OS in patients with mHSPC. As previously mentioned, PSF2 is a prognostic indicator. The PFS2 in group A was significantly longer than that in group B (Fig. 3). Next, we observed the PSA kinetics in both groups. More patients in group A (52/52, 100%) achieved PSA decline more than 90% from baseline (≥ 90% PSA decline) than did patients in group B (44/52, 84.6%) (Fig. 4, p = 0.0428). Furthermore, in group A, PSA decreased by more than 90% from baseline faster than in group B (p = 0.0468). These results suggest that apalutamide prolongs the OS of patients with mHSPC via a deep PSA decline and prolonged PSF2.

## Discussion

In this study, we retrospectively analyzed the data of patients with mHSPCs from our hospital and affiliated hospitals. We found that apalutamide was superior to bicalutamide in terms of PSA-PFS and OS in patients with mHSPC. Furthermore, the PFS2 of mHSPC treated with apalutamide was significantly longer than that of mHSPCs treated with bicalutamide. Patients with mHSPC treated with apalutamide achieved a deep PSA decline (≥ 90% PSA decline) from baseline faster and in larger numbers than those treated with bicalutamide. Among the patients treated with apalutamide, 12 (20%) had grade 3 skin disorders (Supplementary Table 2). Because apalutamide binds weakly to central nervous system-based GABA_A_ receptor, it could cause seizure leading to discontinuation of the apalutamide^13^. Furthermore, apalutamide showed overall 24% severe adverse events while bicalutamide only a few. Although in most cases, the adverse events were controllable by dose reduction, we should keep these adverse events in mind when administering apalutamide. Dose adjustments according to body size may be effective in preventing adverse event from the previous reports^14^.

Globally, upfront ARAT therapy is the mainstream treatment for patients treatment. However, in Japan bicalutamide is commonly used for patients with mHSPC even now, probably due to the reason that Japanese patients with mHSPC respond to bicalutamide better than do other races^15,16^. The differences in responses to bicalutamide between races could be due to genetic, dietary, or environmental factors^17^. Another possible explanation for the difference in responses may be the differences in the administered dosages of bicalutamide between countries^18^. In Japan, where bicalutamide is widely used for the treatment above, an understanding of the relative effectiveness of bicalutamide and ARATs is crucial. Previously, we reported that abiraterone acetate was superior to bicalutamide for mHSPC treatment^19^.

PSA kinetics and the rate and degree of PSA decline after drug administration are associated with long-term outcomes such as OS^6,7,9,10^ in patients with mHSPC treated with the drug. Achieving ≥ 90% PSA decline is associated with better outcome of OS^10^. It is reported that apalutamide may achieve ≥ 90% PSA decline from baseline faster and in larger numbers than does enzalutamide. In this study, we found that apalutamide achieved ≥ 90% PSA decline from baseline faster and in larger numbers than did bicalutamide. It is reported that PFS in patients with mHSPC varies depend on ARATs with which they are treated and apalutamide tends to show better PFS compared to abiraterone^20^. A comparison of the PSA kinetics of apalutamide with those of other ARATs, such as abiraterone acetate, may be required.

In this study, we compared the efficacy of apalutamide and bicalutamide in combination with ADT in patients with mHSPCs in Japan. Although several reports have compared the efficacy of ARAT, which includes apalutamide, abiraterone, and enzalutamide, with bicalutamide in high-risk or high-volume patients with mHSPC^4,5^, this is the first report to directly compare the efficacy of apalutamide with that of bicalutamide in patients with mHSPC, including all risk groups. However, this study has some limitations. The cohort of patients was small and the observation period was short, especially in patients treated with apalutamide. Therefore, a prospective study with a larger cohort over a longer period is required. A prospective study using larger cohort over a longer period is our future work. Previously, we reported that the presence of Gleason pattern 5 (GS5) in the primary lesion may be a predictive factor for the efficacy of abiraterone acetate, another type of ARATs, in high-risk patients with mHSPC^21^. Investigating the difference in the efficacy of apalutamide between patients with mHSPC with GS5 at the primary lesion and those without GS5 in a larger cohort, is a topic for future research.

In conclusion, we suggest that apalutamide combined with ADT is superior to CAB therapy, in terms of OS and PSA-PFS, in patients with mHSPCs. These findings provide useful information for Japanese clinical practice regarding patients with mHSPC.

### Supplementary Information

Supplementary Tables.

## Data Availability

The datasets used and analysed during the current study available from the corresponding author on reasonable request.
