# Supplementary material for: Apalutamide versus bicalutamide in combination with androgen deprivation therapy for metastatic hormone sensitive prostate cancer
Source: Sci Rep. 2024 Jan 6;14:705. doi: 10.1038/s41598-024-51389-w (PMC10771454; doi:10.1038/s41598-024-51389-w)
Supplement: Supplementary file 1 — Supplementary Tables. [file 41598_2024_51389_MOESM1_ESM.docx]

**Apalutamide versus bicalutamide in combination with androgen deprivation therapy for metastatic hormone sensitive prostate cancer**

Takashi Ueda^1^**^*^** MD, PhD, Takumi Shiraishi^1^, MD, PhD, Masatsugu Miyashita^1^, MD, PhD, Naruhiro Kayukawa^1^, MD, PhD, Yusuke Gabata^1^, MD, Satoshi Sako^1^, MD, Ryota Ogura^1^, MD, Atsuko Fujihara^1^, MD, PhD, Koji Okihara^2^ MD, PhD and Osamu Ukimura^1^ MD, PhD

^1^Department of Urology, Kyoto prefectural university of medicine, Kyoto-City, Kyoto 602-8566, Japan.

^2^ Department of Urology, Maizuru Kyosai Hospital, Maizuru-city, Kyoto 625-8585, Japan.

**^*^ Corresponding author.**

Address: Department of Urology, Graduate School of Medical Science, Kyoto Prefectural University of Medicine, Kyoto-City, Kyoto 602-8566, Japan.

FAX: +81 75 251 5598

TEL: +81 75 251 5595

E-mail: t-ueda@koto.kpu-m.ac.jp

**Keywords:** apalutamide, bicalutamide, metastatic hormone sensitive prostate cancer, second progression-free survival, prostate-specific antigen kinetics

Supporting Information

Supplementary Table 1. Characteristics of the overall cohort

Supplementary Table 2. Adverse events in each group.

Supplementary Table 3. Sequential therapies after PSA progression in each group. PSA,

Supplementary Table 1. Characteristics of the overall cohort.

| Hormone therapy |  | Apalutamid + ADT　(group A) | Bicalutamide + ADT (group B) | A vs B |
| --- | --- | --- | --- | --- |
|  |  | n=61 | n=269 | p-value |
| Median age at diagnosis years (range) |  | 77 (61–87) | 77 (51–95) | 0.8375 |
| Performance status (ECOG) | 0 | 44 | 165 | 0.4231 |
|  | 1 | 11 | 58 |  |
|  | 2 | 5 | 19 |  |
|  | 3 | 1 | 12 |  |
|  | 4 | 0 | 2 |  |
|  | not applicable | 0 | 13 |  |
| Median pretreatment PSA level ng/mL |  | 91.9(1.26–7756) | 190.6(4.51–32548) | 0.1591 |
| Median pretreatment ALP |  | 117.5 (56–1682) | 355 (53–8969) | 0.0032 |
| Pathological diagnosis | Gleason score 6 | 2 | 0 | 0.3639 |
|  | Gleason score 7 | 5 | 20 |  |
|  | Gleason score 8 | 19 | 82 |  |
|  | Gleason score 9 | 28 | 141 |  |
|  | Gleason score 10 | 6 | 21 |  |
| Presence of bone metastasis | Yes | 49 | 226 | 0.6520 |
|  | No | 10 | 36 |  |
|  | not applicable | 2 | 2 |  |
| Presence of visceral metastasis | Yes | 17 | 51 | 0.1203 |
|  | No | 44 | 218 |  |
| Presence of Lymph node metastasis | Yes | 33 | 176 | 0.00901 |
|  | No | 28 | 92 |  |
| Median observation period month (range) |  | 15(3-35) | 35(3-261) | <0.0001 |

ECOG, Eastern Cooperative Oncology Group; PSA, prostate-specific antigen; ALP, alkaline phosphatase; ADT, androgen deprivation therapy.

Supplementary Table 2. Adverse events in each group.

| Adverse event  (Group A) | Grade 1 | | Grade 2 | | Grade 3 | | Grade 4 | |
| --- | --- | --- | --- | --- | --- | --- | --- | --- |
|  | No | % | No | % | No | % | No | % |
| Overall | 3 | 5 | 3 | 5 | 14 | 24 | 1 | 2 |
| AST/ALT elevation | 0 | 0 | 0 | 0 | 1 | 2 | 0 | 0 |
| Skin disorder | 2 | 3 | 2 | 3 | 12 | 20 | 1 | 2 |
| Fatigue | 1 | 2 | 0 | 0 | 0 |  | 0 | 0 |
| Interstitial pneumonia | 0 | 0 | 0 | 0 | 1 | 2 | 0 | 0 |
| cough | 0 | 0 | 0 | 0 | 0 | 0 | 0 | 0 |
| diarrhea | 0 | 0 | 1 | 2 | 0 | 0 | 0 | 0 |

| Adverse event  (Group B) | Grade 1 | | Grade 2 | | Grade 3 | | Grade 4 | |
| --- | --- | --- | --- | --- | --- | --- | --- | --- |
|  | No | % | No | % | No | % | No | % |
| Overall | 0 | 0 | 1 | 0.3 | 2 | 0.6 | 0 | 0 |
| AST/ALT elevation | 0 | 0 | 0 | 0 | 1 | 0.3 | 0 | 0 |
| Skin disorder | 0 | 0 | 1 | 0.3 | 1 | 0.3 | 0 | 0 |

Supplementary Table 3. Sequential therapies after progression of disease in each group.

| Agent | **Group A** | **Group B** |
| --- | --- | --- |
|  | **n=8 (13%)** | **n=156 (58%)** |
| **Enzalutamide** | **2** | **64** |
| **Docetaxel** | **4** | **15** |
| **Abiraterone acetate plus prednisone** | **2** | **59** |
| **Dexamethazone** | **0** | **4** |
| **Others** | **3** | **14** |
